# Novel single-chain antibodies against highly conserved epitopes in the hemagglutinin of influenza A viruses: Promising agents for universal therapies 

**DOI:** 10.22038/ijbms.2020.43508.10219

**Published:** 2021-01

**Authors:** Samaneh Alizadeh, Setareh Moazen, Seyed Nooreddin Faraji, Afagh Moattari, Foroogh Nejatollahi

**Affiliations:** 1 Recombinant Antibody Laboratory, Department of Immunology, Shiraz University of Medical Sciences, Shiraz, Iran; 2 Faculty of Pharmaceutical Sciences, University of British Columbia, Vancouver BC, Canada; 3 Department of Bacteriology and Virology, Shiraz University of Medical Sciences, Shiraz, Iran; 4 HIV/AIDS Research Center, Institute of Health, Shiraz University of Medical Sciences, Shiraz, Iran

**Keywords:** Epitope design, Hemagglutinin, Immunotherapy Influenza A virus, Neutralizing scFvs

## Abstract

**Objective(s):**

Development of new antibodies with broad activity would provide anti-influenza prophylaxis and treatment. Human single-chain variable fragments (scFvs) are considered effective agents against viruses. In this study specific human scFvs against highly conserved epitopes in the hemagglutinin (HA) of influenza A viruses were selected and their neutralizing activity was evaluated.

**Materials and Methods:**

Bioinformatic methods were used to evaluate HA epitopes. The panning process selected specific clones from a scFv library. PCR and DNA fingerprinting differentiated the common patterns. Soluble forms of scFvs were produced and evaluated using Western blot analysis. The neutralizing effects of anti-HA scFvs were assessed by microneutralization assay using MDCK cells. Real-time PCR was done to determine the exact copy number of the virus following neutralization.

**Results:**

Bioinformatic evaluation confirmed the antigenicity and accessibility of the epitopes. Four specific anti-HA scFvs, scFvs I, II, I’, and II’ were selected. The scFvs neutralized 2009 H1N1 pandemic and 83.34%, 79.17%, 75%, and 62.5% reduction in the virus titers were obtained following treatments with scFv-II′, I, I′, and II, respectively. Real-time PCR demonstrated 98.6%, 95.7%, 95.26%, and 91.19% reductions in virus numbers following neutralization with scFv-II′, I, I′, and II, respectively.

**Conclusion:**

Anti-HA scFvs selected against highly conserved HA of influenza A virus with high neutralizing effects, offer novel human antibodies for prophylaxis and treatment of a wide range of influenza viruses including different subtypes of H1N1, H3N2, and H5N1 influenza A virus. The antibodies have the potential to be used for universal therapy.

## Introduction

Influenza is the most common acute respiratory infection. There are four genera of influenza viruses: A, B, C, and D (1). Influenza A viruses are responsible for pandemic outbreaks of influenza and annual flu epidemics which lead to high morbidity and mortality (2).

The first outbreak of swine H1N1 influenza A virus was reported in 2009 in the United States and Mexico, which spread rapidly and caused a number of deaths worldwide (3). Virulence of the influenza virus is determined by one of its external glycoproteins, hemagglutinin (HA), which is synthesized in the infected cell and subsequently cleaved into two subunits HA1 and HA2 linked together through disulfide bonds (4). HA1 is highly immunogenic and contains major antigenic epitopes of HA (5). Despite sequence variability of HA, conserved sequences were found in diverse subtypes (6-8).

Identification of neutralizing monoclonal antibodies (mAb) against highly conserved epitopes could provide broadly protective immunity. Several studies have shown that anti-influenza antibodies are capable to prevent or treat influenza (6-9). Hu *et al*. demonstrated presence of seven conserved epitopes in the structure of HA1 of the 2009 H1N1 pandemic influenza A virus, which are responsible for swine binding preference (8). Two highly conserved sequences, GKEVLVLWG and EGRMNYYWTLVEP, which were found in the HA of H1N1 pandemic influenza A virus are also identified with similar structure in other subtypes such as H3N2 and H5N1 (7). Moattari *et al.* reported the same epitope sequences in the HA of 2009 H1N1 pandemic in Shiraz. The two sequences were located in amino acids 187-195 and 241-253, respectively (9). Improvement of gene engineering technology has provided single-chain variable fragment (scFv) as a desirable tool for therapeutic and diagnostic purposes in infectious diseases and cancer (10-14). ScFvs which are composed of VH and VL domains have some advantages over full-length mAb including human origin, small size, deep penetration, high affinity, and specificity for target molecule (15, 16). Also, fast and cost-effective production has made these recombinant antibodies attractive agents for immunotherapy purposes (17-19). 

In this study two highly conserved epitopes of HA, GKEVLVLWG and EGRMNYYWTLVEP, were used to select specific anti-HA single-chain antibodies. The antigenicity and accessibility of epitopes were evaluated by bioinformatic methods. The soluble forms of anti-HA scFvs were produced and the neutralizing effects of the specific scFvs against the 2009 H1N1 pandemic influenza A virus (9) were evaluated using microneutralization assay. The effects of antibodies were further assessed by determining the copy numbers of the virus following treatment with the specific anti-HA scFvs by quantitative Real-time PCR assay.

## Materials and Methods

***Homology modeling of HA antigen***

HA of influenza A virus isolated from Iranian patients during the outbreak of 2009 pandemic influenza A (H1N1) virus infection, A/Shiraz/8/2010 H1N1 (9), was applied for homology modeling using the swiss-model server at https://swissmodel.expasy.org/. Influenza hemagglutinin antigen (6n41.1.B) was used as the template. Following modeling, the 3D structure of the HA antigen was refined at https://zhanglab.ccmb.med.umich.edu/ModRefiner/ and evaluated by RAMPAGE at http://mordred.bioc.cam.ac.uk/~rapper/rampage.php. For visualizing the 3D structures of the HA antigen, Chimera 1.10.2 software was used. 

***Evaluation of epitopes***

To evaluate the antigenicity and accessibility of conserved HA epitopes, GKEVLVLWG and EGRMNYYWTLVEP, the hemagglutinin antigen from influenza A virus (9) was used. To investigate the most antigenic epitope, IEDB server at https://www.iedb.org/, and to predict linear accessible epitopes, SVMTriP program at http://sysbio.unl.edu/SVMTriP/ were utilized. The suggested peptides were investigated on the 3D structure of modeled HA. BLAST of the selected epitopes was carried out on the BALSTP server at https://blast.ncbi.nlm.nih.gov/Blast.cgi to assess the cross-reactivity with other antigenic peptides. The confirmed epitope sequences were synthesized by the TAG Company (Copenhagen, Denmark) with > 85% purity.

***Phage rescue***

Phage rescue was performed by helper phage M13 KO7 (BioLabs, New England) using a scFv library as previously described (20). Phage transformed *Escherichia coli* bacteria (*E. coli*) were grown overnight at 30 °C on a 2TYG agar/ampicillin plate. After scraping the cells into 50 ml 2TYG broth media, the culture media was shaken at 37 °C for 1 hr. *M13KO7* was added and incubated at 37 °C for 30 min. The culture media was centrifuged and the pellet was transferred into 50 ml 2TY broth containing ampicillin and kanamycin, and incubated overnight with shaking at 30 °C. The supernatant was collected following centrifugation, filtered using 0.22 μm filters (Orange, Belgium), and stored at 4 °C. 

***Selection of specific scFvs***

Immuno-tubes (Nunc, Roskilde, Denmark) were coated with each HA peptide at 4 °C overnight. After washing with PBS, blocking buffer, 2% skimmed milk was added to the tubes and incubated at 37 °C for 2 hr. Following washing with PBST and PBS, the diluted phage-rescued supernatant (10^12^ PFU/ ml) was added and incubated for 1 hr at room temperature. Following elution by *E. coli* *TG1* cells, the obtained clones were rescued and four rounds of panning were performed to select specific anti-HA scFvs.

***PCR and DNA fingerprinting***

PCR and DNA fingerprinting of randomly selected clones were done to determine the common patterns with high frequency after the fourth round of panning. Following PCR ampliﬁcation of the scFv inserts (30 cycles of 94 °C for 1 min, 55 °C for 1 min, and 72 °C for 2 min using universal R1 and R2 primers R1: CCATGATTACGCCAAGCTTTGGAGCC, R2: CGATCTAAAGTTTTGTCGTCTTTCC), PCR products were digested using Mva I restriction enzyme (Fermentas, Lithuania) at 37 °C for 2 hr. 

***Expression and extraction of soluble scFvs***

To produce soluble scFvs, recombinant phages from selected clones, clones I, II, I´ and II´, against peptides 1 and 2 were used to infect log phase *HB2151* *E. coli*. The infected cells were cultured in 2TY media supplemented with ampicillin at 30 °C with shaking. 1 mM IPTG as an inducer was added to the infected *HB2151* *E. coli* and incubated overnight at 30 °C. Following centrifugation, the bacterial pellet was collected and used for the periplasmic extract of scFv. The pellet was resuspended in lysis buffer (30 mM Tris Hcl+100 mM NaCl+100 Mm Na_2_HPO_4_+8 Mm urea, PH: 8) and incubated on ice for 1 hr with shaking. The bacteria were sonicated for 5×1 min. Following centrifugation, the supernatant containing soluble scFv was filtered using Ultra-15 centrifugal filter units (Amicon, USA, 10000 KDa) and Ultra-0.5 centrifugal filter units (Amicon, USA, 30 KDa). The concentration of the purified scFvs was determined using Bradford assay (Bio-Rad, Ontario, CA) and equal concentrations of scFvs were prepared for further assays.

***Western blotting***

Western blotting was done to confirm the presence of the soluble scFvs in periplasmic extracts. The SDS reducing buffer (25 M Tris Hcl, 15% SDS, 50% Glycerol, 25% 2ME, 0.01% bromophenol blue) was added to 10 μg/ml of each scFv periplasmic extract and heated to

95 °C for 8 min. The mixture was run through a 6% stacking and 10% running gel and the separated proteins were transferred onto a PVDF membrane (Millipore, Bedford, MA, USA). After blocking by 5% skimmed milk, anti-c-myc antibody (Sigma, Germany) was added and incubated at 37 °C for 2 hr. Following washing, HRP-conjugated anti-rabbit antibody (Sigma, Germany) was added and the test was developed using Bio-Rad chemiluminescence kit (Bio-Rad Laboratories, USA).

***Microneutralization assay***

The neutralizing activity of anti-HA soluble antibodies was evaluated by microneutralization assay. Briefly, 100 TCID50 of the virus was pre-incubated with each scFv antibody (5.5 mg/ml) at 37 °C for 1 hr. The mixture was used to infect confluent MDCK cells (Pasteur institute, Iran) in a microplate. The MDCK cells treated with the virus were considered as the positive control and MDCK cells without any treatment were considered as negative control. The plate was incubated for 72 hr. Infectivity of the virus was identified by the presence of cytopathic effects (CPE) and the titer was calculated using the Reed and Muench method (21). The test was performed four times and the contents of the wells were combined.

***Quantitative real-time PCR***

RNA was extracted from infected MDCK cells and control samples obtained from microneutralization assay, using a viral RNA extraction kit (Roch, Mannheim, Germany). Real-time PCR was performed using Swine H1N1 influenza human pandemic strain Real-time kit (Primer design, USA). The extracted RNA (5 μl) was added to the master mix. The qPCR was performed as follows: reverse transcription at 55 °C for 10 min, enzyme activation at 95 °C for 8 min, and 50 cycles at 95 °C for 10 sec. Following amplification, CT (cycle threshold) values of samples were compared in order to assess differences in mRNA levels of the target genes. Data acquisition and analysis of the real-time PCR assays were accomplished using the Step One Analysis Software, version 2.3 (Applied Biosystems, USA). The following formula was used:

Changes in copy number after treatment = copy number of control – copy number after treatment/copy number of control x 100

## Results

***Epitope evaluation***

**Figure 1:**
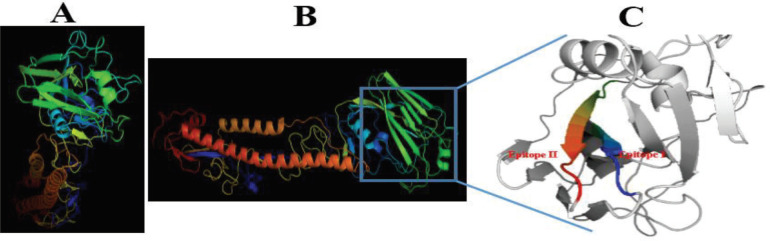
Three-dimensional structure of hemagglutinin (HA) antigen sequence from 2009 H1N1 pandemic influenza A virus using modeler software. Top view (A) and bottom view (B) of the HA antigen are shown. The HA antigen with selected epitopes, epitopes 1 and 2 (C)

HA of influenza A/Shiraz/8/2010 H1N1 (9) was modeled based on 6n41.1.B protein and showed 79.67% sequence identity and 87% coverage with the template. GMQE and QMEAN were 0.79 and -0.55, respectively. Among the output sequences resulting from IEDB and SVMTriP servers, the epitope sequences including GKEVLVLWG and EGRMNYYWTLVEP, epitopes I and II from amino acids 187-195 and 241-253 of the HA antigen were confirmed as immunodominant epitopes based on criteria including accessibility and antigenicity. The modeled 3D structure of the HA antigen as well as the selected epitopes are shown in Figure 1. Blasting the selected epitopes showed no homology with human antigens. 

***Selection of anti-HA scFvs***

**Figure 2:**
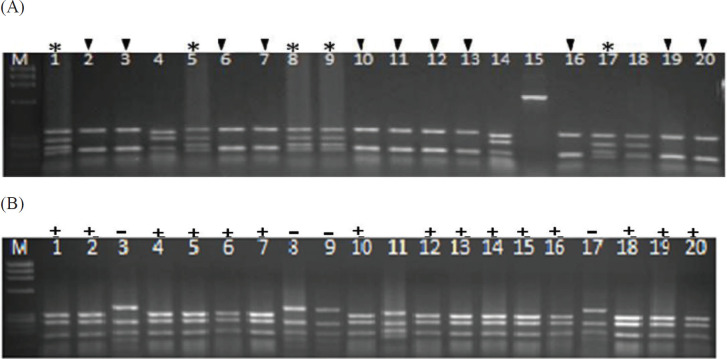
DNA fingerprinting of 20 selected single-chain variable fragment (scFv) clones. Common patterns with frequencies of 25% (lanes 1, 5, 8, 9, 17) and 55% (lanes 2, 3, 6, 7, 10, 11, 12, 13, 16, 19, 20) were obtained against peptide 1 (A), and two patterns with frequencies of 20% (lanes 3, 8, 9, 17) and 75% (1, 2, 4, 5, 6, 7, 10, 12, 13, 14, 15, 16, 18, 19, 20) were obtained against peptide 2 (B)

Figures 2 A and 2 B show DNA fingerprinting of clones obtained after four rounds of panning against peptides 1 and 2, respectively. Two common patterns were obtained against each peptide containing scFv-I and svFv-II against peptide 1 and scFv- I’ and scFv- II’ against peptide 2. The frequencies of the selected scFvs were 25%, 55%, 20%, and 75%, respectively.

***Western blotting analysis***

**Figure 3:**
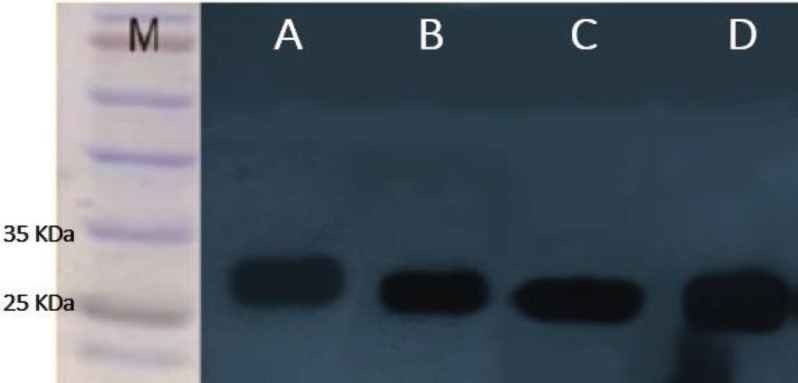
Western blot analysis of the soluble single-chain variable fragment (scFvs) from periplasmic extracts. scFv-I (A), scFv-II (B), scFv-I′ (C), and scFv-II′ (D)

Following expression of soluble scFvs, Western blot analysis was performed to confirm the expression of the antibodies. Figure 3 showed ~27 KD bands in the periplasmic extract of *HB2151* *E. coli* bacteria after induction by 1 mM IPTG.

***Neutralization activity***

As shown in Table 1, soluble scFv-II′ antibody showed the minimum infectious titer, 1 mean log_10_ TCID50/ml. Infectious titers of soluble scFv-I and scFv-I′ antibodies were 1.25 and 1.5 mean log_10_ TCID50/ml, respectively. Maximum infectious titer, 2.25 mean log_10_ TCID50/ml, was obtained when the virus was treated with scFv-II antibody. The virus titer in virus control without addition of antibody was 6 mean log_10_ TCID50/ml.

***Quantitative real-time PCR***

**Figure 4:**
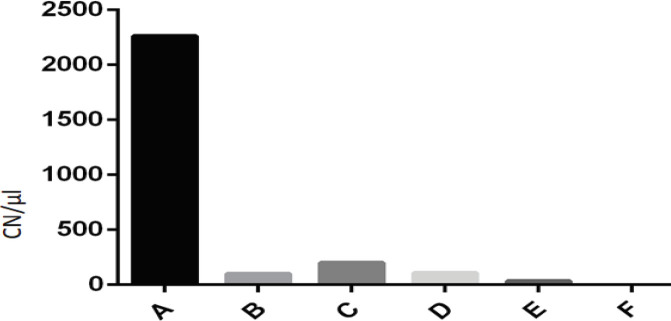
Results of real-time PCR. Copy number of the virus in 1 µl of RNA extract (CN/μl) following neutralization from MDCK cells: in virus control, no Ab treated, (A), treated with soluble scFv-I antibody (B), treated with scFv-II antibody (C), treated with scFv-I' antibody (D), treated with scFv-II' antibody (E), and in cell control, MDCK cells without virus, (F)

Following RNA extraction, Real-time PCR was performed to determine the exact copy number of the virus following neutralization by the four specific anti-HA scFv antibodies (Figure 4). The virus copy numbers which were obtained from RNA extracts (5 μl) were presented as CN/ μl. Treatment of the virus with soluble scFv-II’ antibody had the lowest copy number of the virus, 32 copy number/μl (CN/ μl). The copy numbers of the virus after treatment with soluble scFv-I and scFv-I’ antibodies were 96 and 107 CN/μl, respectively. Maximum copy numbers, 199 CN/μl, of the virus was obtained after treating the virus with the scFv-II antibody. The copy number of virus control with no antibody treatment was 2259 CN/μl.

**Table 1:** Microneutralization assay results. Infectious titers of the virus after treating with soluble single-chain variable fragment (scFv) I, II, I´and II´. The virus control contained virus without antibody

**Soluble antibodies**	**Mean log**_10_ **TCID50/ml**
1. scFv-I	1.25
2. scFv-II	2.25
3. scFv-I'	1.5
4. scFv-II'	1
5. no Ab (virus control)	6

**Figure 5:**
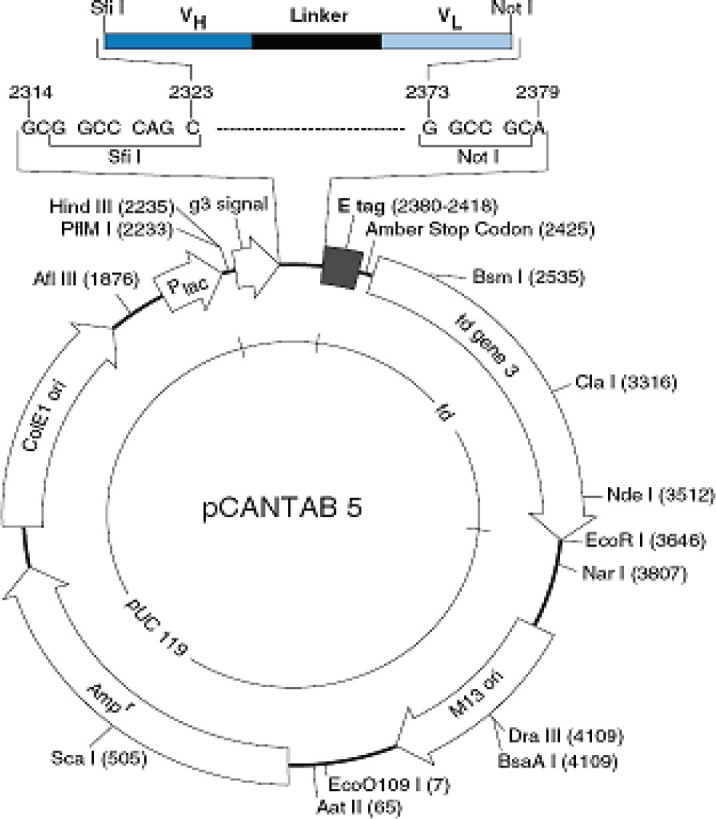
Genetic map of pCANTAB 5 phagemid vector. scFv gene, amber stop codon, and fd gene3 of recombinant phage are shown (22)

## Discussion

Passive immunization using neutralizing antibodies could provide protection against influenza infection. Studies in animals demonstrated the prophylactic and therapeutic efficacy of neutralizing antibodies against influenza infection and have shown the effective role of the antibodies in comparison with antiviral drugs (23). As the broadly neutralizing antibodies have the capability to bind to diverse strains of influenza, they could be the best treatment option in the influenza pandemic along with vaccine development. The antibodies can also have an effective role in seasonal influenza for treatment of severe acute cases (24). Human mAbs, prepared from patients with influenza infection, induced broad neutralization and provided information regarding important human epitopes for both development of vaccine and neutralizing antibodies (6). The conserved epitopes of HA have been introduced as ideal targets for producing broad-acting neutralizing antibodies (6, 7). 

In this study, the three-dimensional structure of the HA protein from H1N1 influenza A virus was modeled based on homology modeling and the antigenicity and accessibility of the two highly conserved epitopes were evaluated and confirmed. Homology modeling is a common tool for interpretation of protein structure and function and reveals the 3D conformation of a protein and shows its binding activity (18, 25, 26). The two highly conserved epitopes in 2009 swine H1N1 pandemic are also highly conserved in human H1N1, human H5N1, and avian H5N1. The epitopes are fairly conserved in human H3N2, swine H1N1, and swine H3N2 (7). As shown in Figure 1 the epitopes are exposed and will be accessible for binding with antibodies. The results of blasting showed no homology between the epitopes and human antigens and demonstrated that the antibodies against these epitopes would not cross-react with human tissues and can be used for influenza targeted therapy. 

Phage rescue procedure provided phage antibodies with scFvs displayed on minor coat proteins. The obtained antibodies were used for the panning process. Following four rounds of panning, fingerprinting results showed common patterns with 55% and 25% frequencies against epitope 1, and 75% and 20% frequencies against epitope 2, and therefore four specific anti-HA scFvs were selected. Some studies have shown the effectiveness of the panning process in the selection of specific and active antibodies. A specific scFv against conserved M_2 _protein of H5N1 was selected by the panning process which inhibited replication of different strains of H5N1 influenza A virus and reduced the number of intracellular viruses in the infected cells (27). Also, in a similar study, a specific scFv, 4F5, was selected against recombinant HA1 of the influenza virus. The selected antibody could effectively neutralize different clades of H5N1 influenza A virus and reacted with a conserved sequence in HA1 (28).

Soluble forms of selected scFv antibodies were produced using *HB2151* *E. coli* bacteria. Non-suppressing *HB2151* *bacteria* recognizes an amber stop codon located between the scFv and gene III fragment in the *pCANTAB 5* vector, prevents the expression of an scFv-g3p fusion protein, and allows the expression of an scFv as a soluble fragment, Figure 5 (22, 29). 

Western blotting confirmed the expression of four anti-HA scFvs. The neutralizing effects of the soluble anti-HA scFvs, I, II, I’ and II’, against the 2009 H1N1 pandemic influenza A virus isolated during the human swine flu outbreak of 2009-2010 in Shiraz (9), were evaluated in microneutralization assay. The results demonstrated that all of the selected scFvs neutralized the virus. ScFv-II’ antibody showed the highest neutralizing activity. The titer of the virus treated with scFv-II’ was 1 mean log_10_ TCID_50_/ml which represented 83.34% neutralization effect compared with virus control. The virus titers obtained after treatment with scFv-I, scFv-I’ and scFv-II were 1.25, 1.5, and 2.25 mean log_10_ TCID_50_/ demonstrating 79.17%, 75%, and 62.5% neutralization compared with the virus control, respectively. Specific scFv antibody against protein G of rabies virus demonstrated a highly viral neutralizing activity and enhanced neutralizing potency *in vivo* (30). Neutralizing antibodies targeting HA2 of influenza virus suppressed infections with H5N1 and H1N1 viruses *in vitro* (31). According to Teferedegne *et al*. antisera containing neutralizing antibodies against the influenza virus could successfully neutralize the virus 95-100%, and at least 5 log_10_ reductions were observed (32). Similarly, we detected 5 log_10_ reductions in TCID50/ml for scFv-II’ when compared with virus control. Although the virus neutralization test can determine the efficiency of antibodies in virus neutralizing, the virus-cell attachment stage is often the limiting factor that may affect the results.

 Real-time PCR was done to quantify the viral copy number (CN) after treatment of the virus with specific anti-HA scFv antibodies. The least CN of the virus, 32 CN/μl, was obtained after treatment with soluble scFv-II’ antibody, which represented a 98.6% reduction in the virus CN compared with the titer of virus control, 2259 CN/μl. The CNs of the virus after treatment with scFv-I, scFv-I’, and scFv-II were 96, 107, and 199 CN/μl, respectively, indicating 95.7%, 95.26%, and 91.19% reduction in virus CN in comparison with the CN of the virus control. Although the results of real-time PCR confirmed the results obtained in the microneutralization assay, the higher percentages in Real-time PCR could be due to its higher sensitivity. Real-time PCR is widely considered an ideal tool for detecting the viral load and exact CN of viruses due to its high specificity and sensitivity, quantitative measurement, and standardization (33, 34). The test is based on molecular assessments of the genome and is more accurate than phenotypic tests therefore small changes are detectable (35). In order to detect the viral load of viruses in various samples, real time PCR demonstrated an ideal detection system (36-39). Ellis *et al.* (33) applied real-time PCR assay to report the exact copy number of influenza A and influenza B viruses in specimens of patients with respiratory infection. Although real-time PCR is defined as an accurate test, increasing variation with cycle number is a limitation of such a study.

Although antiviral drugs and vaccination provide treatment and prophylaxis strategies against influenza infection, influenza infection remains a noteworthy public health concern. Antibody therapy has been effective in reducing viral load during virus infection, especially in high-risk individuals such as immunocompromised patients (40, 41). A broadly neutralizing influenza HA stem-specific monoclonal antibody, CR8020, and also a monoclonal antibody against the conserved helical region in the membrane-proximal stem of HA1 and HA2, CR6261, are in phase I clinical trials and have shown promising results (42, 38). However, due to the possibility of HAMA reaction, fully human scFvs are not immunogenic and more efficient antibodies for clinical applications (43). 

The selected neutralizing scFvs have the potential to be used for passive immunization against influenza A viruses.

The properties of scFvs including human origin, small size, high affinity and specificity, effective tissue penetration, and the ability to be manipulated by genetic engineering (13, 15, 16, 18) suggest additional therapeutic benefits of the neutralizing anti-HA scFvs. Further investigations are needed to evaluate the effects of the selected anti-HA antibodies *in vivo*.

## Conclusion

The neutralizing scFvs selected against highly conserved sequences of HA with high neutralizing effects offer novel human antibodies with broad activity that could prevent escaping of a wide range of influenza viruses including H1N1, H3N2, and H5N1 influenza A virus and even newly evolved viruses. 
